# Advancements and Challenges of Artificial Intelligence-Assisted Electroencephalography in Epilepsy Management

**DOI:** 10.3390/jcm14124270

**Published:** 2025-06-16

**Authors:** Yujie Chen, Zhujing Ou, Dong Zhou, Xintong Wu

**Affiliations:** Department of Neurology, West China Hospital of Sichuan University, No. 37 Guoxue Alley, Wuhou District, Chengdu 610041, China; chenyujie123@stu.scu.edu.cn (Y.C.); 15008288672@163.com (Z.O.); zhoudong66@yahoo.de (D.Z.)

**Keywords:** electroencephalography, epilepsy, machine leaning, deep learning

## Abstract

Artificial intelligence (AI) has emerged as a transformative tool in the analysis and management of epilepsy through its integration with electroencephalography (EEG) data. The adoption of AI-assisted solutions in managing epilepsy holds the potential to significantly enhance the efficiency and accuracy for diagnosing this complex condition. However, AI-assisted EEG technologies are infrequently adopted in clinical settings. In this Review, we provide an overview of AI applications in seizure prediction, detection, syndrome classification, surgical planning, and prognosis prediction. Additionally, we explore the methodological considerations and challenges that are relevant in clinical settings. Overall, AI has the potential to revolutionize epilepsy management, ultimately improving patient outcomes and advancing the field of precision medicine. Fostering interdisciplinary collaborations between AI researchers, neurologists, and ethicists will be crucial in creating integrated solutions that address both technical and clinical requirements.

## 1. Introduction

Epilepsy is one of the most common neurological disorders. Globally, approximately 50 million individuals are affected by epilepsy. The clinical management of epilepsy focuses on three primary objectives: preventing the onset of the disorder, accurately diagnosing epilepsy versus similar conditions, and developing effective treatment strategies. Achieving these goals requires the comprehensive analysis of complex data, including patient medical histories, video recordings, electroencephalography (EEG), magnetic resonance imaging (MRI), and information from wearable technologies [1]. EEG is a crucial tool for recording and monitoring brain electrical activity, primarily derived from postsynaptic potentials. Over the years, EEG has become an essential tool in the field of epilepsy, playing a critical role in diagnosing seizures and guiding treatment strategies. The methods of EEG recording can be divided into the following: portable/dynamic EEG, video EEG (vEEG) and intracranial EEG (iEEG) with specific applications like neonatal EEG, which holds unique clinical significance. Despite its widespread use, the manual examination of these signals by experts is strenuous and time-consuming, as is the case with long-term recordings, and EEG signals are highly dynamic in nature. Due to this randomness, the neurologists may sometimes give inconsistent results which may in turn affect the performance of detecting epileptic seizures, limiting efficiency and accuracy [2]. Hence, artificial intelligence (AI) techniques can be used to extract nonlinear statistical regularities for improving the accuracy of detection. The application of AI technologies in human brain research has increased. This review aims to summarize recent advancements in AI-assisted EEG applications in the field of epilepsy and to discuss the challenges that remain.

## 2. Methodology

A scoping review was conducted to map the existing literature on AI-assisted EEG analysis in EEG-based epilepsy management. The methodology aligns with the PRISMA guidelines and emphasizes transparency in study identification, screening, and selection.

### 2.1. Identifying the Research Question

This review aimed to explore the scope and roles of AI in epilepsy diagnosis and treatment using EEG data. The research questions were refined as follows:

① What types of AI models are utilized for EEG analysis in epilepsy?
② What are the roles of AI systems in epilepsy management?
③ What are the challenges in implementing AI for EEG-driven epilepsy care?

### 2.2. Search Strategy

Two investigators independently searched PubMed, IEEE Xplore, Web of Science, and Google Scholar using a combination of Medical Subject Headings (MeSH) and free-text terms. Key search terms included:

For Epilepsy terms, a combination of the words “epilepsy” [MeSH] OR “seizure” [Title/Abstract] OR [Title/Abstract] OR “epilep*” [Title/Abstract] were used.

For AI terms, a combination of the words “artificial intelligence” [MeSH] OR “machine learning” [Title/Abstract] OR “deep learning” [Title/Abstract] OR “convolutional neural network” [Title/Abstract] OR “artificial neural network” [Title/Abstract] OR “machine intelligence” [Title/Abstract] were used.

For EEG terms, a combination of the words “Electroencephalography” [MeSH] OR “EEG” [Title/Abstract] OR “Electroencephalogram” [Title/Abstract] was used.

### 2.3. Inclusion and Exclusion Criteria

Inclusion criteria:

① Study type: Randomized controlled trials, pilot studies, pre-post trials, quasi-experiments, cross-over trials, observational studies, qualitative studies, and mixed-method studies.
② Language: English or Chinese publications.
③ Participants: Patients diagnosed with epilepsy.
④ Intervention: AI models (e.g., machine learning, deep learning) applied to EEG data for seizure management like seizure detection, prediction.
⑤ Outcomes: performance metrics.

Exclusion criteria:

① Non-eligible publication types: reviews, conference papers, case reports, letters, and animal studies.
② Studies older than 10 years.
③ Irrelevant topics (e.g., non-AI/EEG applications, non-epilepsy research).
④ Full-text unavailable or insufficient methodological details.

Two authors independently screened titles and abstracts to identify eligible studies. The full texts of these studies were assessed based on the inclusion criteria. Discrepancies were resolved by discussion among the authors. Given the exponential growth of artificial intelligence (AI) research in recent years and the prevalence of methodological redundancy across studies, we implemented stringent inclusion criteria to prioritize high-quality articles for this scoping review. Selected publications were required to meet at least one of the following benchmarks: (1) a citation count of ≥30 in the Web of Science (WOS) core database, indicating sustained academic influence; (2) publication within the past year in journals ranked in the top quartile or equivalent by recognized metrics. This multi-dimensional approach ensures the inclusion of seminal, timely, and methodologically robust works, thereby enhancing the review’s validity and relevance to contemporary advancements in AI. However, a formal quality assessment of the included studies was omitted due to the nascent stage of AI applications in EEG management, where most research comprises preliminary, small-scale exploratory investigations.

## 3. Characteristics of the Included Studies

**Figure 1:**
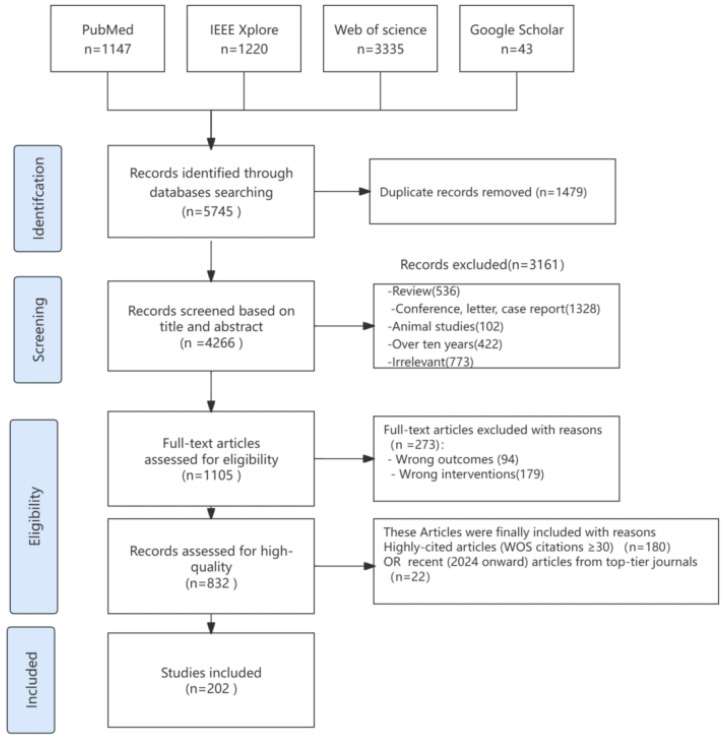
PRISMA flowchart of study identification, screening, and selection.

**Figure 2:**
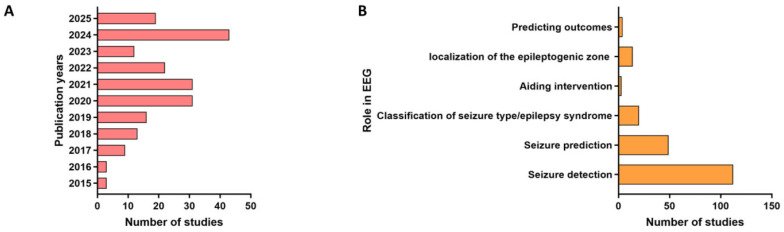
Flowchart illustrating the identification, screening, and inclusion process of studies. (**A**) Annual distribution of included studies (2015–2025). (**B**) Roles of EEG applications in epilepsy.

The study selection process, outlined in Figure 1, followed the PRISMA framework. A total of 202 studies meeting predefined inclusion criteria (e.g., high citation impact [WoS citations ≥ 30] or recent publications from top-tier journals) were included. Annual publication trends (Figure 2A) demonstrate a substantial surge in research output from 2015 to 2023, with a notable acceleration in recent years (e.g., 40 studies in 2023 versus 12 in 2018), reflecting intensified scholarly interest in AI-driven EEG applications. To answer the question of what AI technologies are currently employed to support EEG management in epilepsy (Figure 2B); the majority of those studies targeted seizure detection (112 studies, 55.4%), followed by seizure prediction (49, 24.3%), epilepsy syndrome classification (20, 9.9%), and epileptogenic zone localization (14, 6.9%). A smaller subset explored intervention outcome prediction (4 studies) and EEG-guided clinical interventions (three studies). Detailed methodological and categorical characteristics of the included studies are summarized in Figure 2.

### 3.1. What Is Artificial Intelligence and How Did It Work?

**Figure 3:**
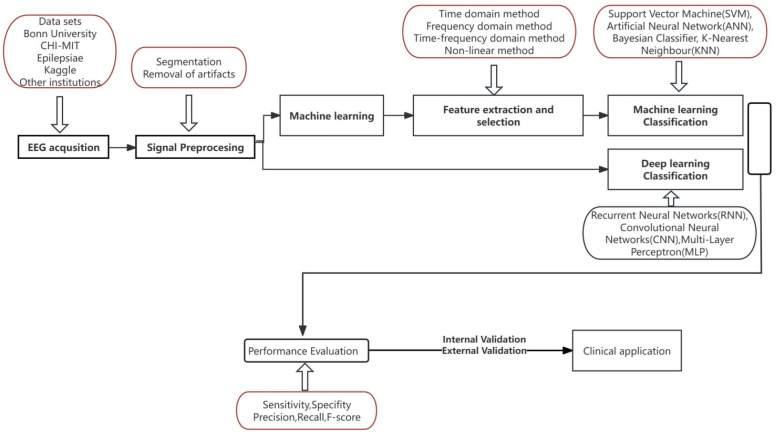
Methodological pipeline for EEG data analysis using AI techniques. Solid arrows in this diagram denote listing relationships.

Building a model involves multiple steps, which are EEG data acquisition, data preprocessing, development of a machine learning or deep learning model, and a final performance evaluation step. Time, frequency, time-frequency domain features, and nonlinear characteristics are the most representative EEG features, with raw EEG signals decomposed using these analyses for feature extraction and classification [3,4]. EEG data are characterized by noise, nonlinearity, and non-stationarity, reflecting the challenges posed by external interference, complex neural dynamics, and time-varying signal properties. Hence, nonlinear analysis of EEG signals was found to effectively capture complex physiological phenomena, providing valuable insights into brain function, making it a suitable approach for understanding brain disorders and detecting epileptic stages [5]. Classification of EEG signals require efficient methods that are sensitive to noise and outliers [6]. After EEG decomposition, pre-processing and data cleaning, feature extraction methods are a critical step in reducing classification error rates and computational complexity. Although they are diverse and unique to EEG studies, they can be broadly categorized into three main groups: energy-based features, statistical features, and distribution/histogram-based features. Later, a threshold or model-based criteria must be applied to the features to determine the presence or absence of a seizure. This step is called classification. Classification algorithms include machine learning, deep learning, and transfer learning frameworks that may predict a future seizure and offer seizure detection and a specific seizure-type classification [7,8]. Machine learning involves supervised learning (labeled data) and unsupervised learning (pattern detection). With either approach, informative input features are identified in a process termed feature selection (either manually, based on expert-level knowledge, or by the data-driven algorithm), then analyzed using a mapping function that generates output predictions from these features. The huge data availability has also boosted the development of deep learning. A deep learning algorithm is a subset of neural networks where there are multiple layers of processing the input data to predict the variable of interest. Techniques like Support Vector Machines (SVMs), Autoencoders, and Convolutional Neural Networks (CNNs) enable tasks such as classification. Clustering and anomaly detection belonged to unsupervised machine learning algorithm, and CNN-based model is presented instead of the manual feature extraction method which is popular in deep learning algorithm [9,10]. SVM relies on manually crafted features and uses kernel functions to address nonlinear problems by optimizing the maximum margin hyperplane for classification or regression. In contrast, deep learning architecture employs neural networks for automatic feature learning and excels in handling large-scale data and complex tasks, where SVM faces limitations [11,12]. Deep neural networks’ black-box nature compromises algorithm reliability, limiting their large-scale use in medicine [13]. We recognize that high quality dataset, effective pre-processing, feature extraction, and classification are the keys of the AI model, and regularization and heuristic algorithms help balance computational complexity and accuracy, improving predictive performance on new data [14]. The methodological pipeline for EEG data analysis using AI techniques is illustrated in Figure 3, Tables S1 and S2.

### 3.2. Seizure Prediction

Epilepsy is characterized by recurrent seizures that significantly impact quality of life. Accurate prediction of seizure events remains a critical challenge, as timely intervention can enhance patient outcomes and reduce morbidity. EEG recordings can detect sudden alterations in brain electrical activity associated with neurological disorders. This makes it a promising tool for seizure prediction. Epileptic signals are categorized into four phases. The distinction between preictal (prior to seizure) EEG pattern and interictal (between seizures) EEG patterns is substantial within the given training dataset, making it crucial for accurate seizure prediction. Preictal EEG clip data are sampled and featured within one hour before seizure onset in most studies. In the context of model evaluation, Maiwald introduced additional criteria for epilepsy prediction, including the maximum false prediction rate, seizure onset period, and seizure prediction horizon [15]. Balancing false prediction rate and sensitivity is essential, as reducing detection latency is key to preventing seizures [16,17]. Since Gotman’s pioneering study [18] utilizing EEG for the detection of epilepsy, numerous researchers have conducted extensive experiments to advance and refine methodology to predict seizures. Early attempts at seizure prediction utilizing machine learning methods, particularly through the widespread use of SVM, demonstrated promising results with high sensitivity and low false alarm rates [19,20]. The deep learning method has advanced the extraction of information from complex datasets and consistently demonstrated superior performance in classification tasks [17,21,22]. CNNs have emerged as a popular deep learning method for predicting seizures. Yochum et al. used 1D-CNNs to predict absence seizure in children using scalp EEG [23].Wei et al. [24] proposed a seizure prediction method using a compact graph convolutional network with adaptive functional connectivity and CNN-inspired depthwise separable convolutions, achieving higher accuracy and lower complexity compared to traditional CNN-based models. Considering the high-variability in subjects’ EEG data, studies based on subject-independent models usually obtain more robust outcomes compared to those utilizing data that is not independent of the patients [16]. Dissanayake proposed two patient-independent deep learning models reaching state-of-the-art seizure prediction accuracies of 88.81% and 91.54% on the CHB–MIT–EEG dataset [25]. Although patient-independent deep learning models enhance generalization and scalability in epilepsy management, they have limited adaptability to individual patient differences and require more data. Transfer learning modifies a general model into a patient-specific one by fine-tuning it with a small portion of individual recordings, which is found to be customizing personalized models [26]. However, current deep learning models classify signal features separately and overlook the relationships among them, thereby hindering the identification of hidden seizure patterns. Therefore, utilizing hybrid models like CNN–Long Short-Term Memory (LSTM) or hybrid CNN–RNN (Recurrent Neural Network) models leverages the strengths of CNN for extracting spatial features and LSTM networks for modeling temporal dependencies. This integration can simultaneously process spatial and temporal information, leading to more accurate and comprehensive interpretations of brain activity [27,28,29,30,31]. Portable EEG systems are introduced to closely monitor patients’ conditions [21]. However, EEG-based prediction faces inherent challenges: EEG signals are inherently weak and susceptible to environmental noise, and most studies are conducted in controlled experimental settings, potentially limiting model accuracy in real-world applications. Furthermore, wearing EEG electrodes for extended periods can be uncomfortable for patients, and long-term surface electrode recordings may become challenging to interpret because of increasing impedance. Additionally, challenges such as signal variability across patients and the need for large-scale, multicentric datasets remain [32]. Selected models for seizure prediction were shown in Supplementary Table S3.

### 3.3. Seizure Detection

Epilepsy diagnosis as defined by the new 2017 International League Against Epilepsy (ILAE) Seizure Classification involves three steps, starting with classifying seizure type; the second level is to confirm epilepsy type; and the third level is to consider a specific epilepsy syndrome [33]. Both seizure and epilepsy classifications take into account results from diagnostic investigations involving EEG. EEG can be categorized into routine EEG and vEEG. Routine EEG is utilized for differentiating paroxysmal central nervous system disorders, such as epilepsy and sleep-related disturbances as well as for assessing brain function in critically ill patients and evaluating comatose individuals. The examination requires only approximately 10 min; however, its positive detection rate is relatively low, limiting its use to screening purposes. In contrast, long-term vEEG (>24 h) is employed to confirm the diagnosis of a seizure disorder, accurately classify seizure types, and determine seizure origins [34,35]. The appilication of vEEG boasts a high positive detection rate, making it the clinical gold standard for diagnosis. Nevertheless, the interpretation of vEEG data demands hospitalization and the need for special resources and personnel, posing significant challenges in clinical settings. In the recent 10 years, studies have made significant progress in automatic epilepsy detection tasks by employing machine learning and deep learning methods to distinguish between epileptic and non-epileptic seizures [36,37,38,39,40,41], classify seizure types [13,42,43], and determine epilepsy syndromes (in the later text). For example, nonconvulsive status epilepticus is a condition characterized by subtle symptoms, undetected seizures, and persistent EEG epileptic activity. These prolonged seizures can lead to irreversible brain damage, making them a medical emergency [44]. Delays in diagnosing nonconvulsive status epilepticus in emergency departments are frequent. AI detection shows potential as a valuable tool in emergency settings [45]. EEG-based nonconvulsive seizure detection utilizing advanced SVM classifiers for high-precision, achieving over 98% sensitivity and accuracy, adapting to pattern changes, ensuring real-time reliability [46,47]. Encouragingly, SCORE-AI is the first fully automated model for routine EEGs, classifying recordings as epileptiform-focal, epileptiform-generalized, or non-epileptiform-diffuse. Trained on 30,493 expert-annotated EEGs, it matches human experts with 90% specificity [48]. This classification accuracy can reach up to 85%, prompting the use of these innovative techniques to support neurologists in early diagnoses. Integrating video with EEG mitigates movement-induced interference, enhancing interictal epileptiform discharges detection reliability. The multimodal model vEpiNet achieved a precision–recall curve (PR–AUC) of 0.8623 in prospective testing, outperforming the single-modality nEpiNet (0.8316). This highlights the advantage of integrating video data to mitigate artifacts from patient movements and environmental noise. vEpiNet also reduced false positives while maintaining high specificity (94%) and accuracy (92%) in cross-validation. Combined with its resilience to physiological noise (e.g., sleep waves, myoelectric artifacts) and robust area under the receiver operating characteristic curve (0.9902), the precision-recall area under the curve further underscores vEpiNet’s clinical viability for efficient detection of interictal epileptiform discharges in real-world, artifact-prone settings [49]. Integrating video with EEG reduces movement-related interference and distinguishes artifacts, enhancing interictal epileptiform discharges(IED) detection. This is a crucial step toward their implementation in clinical practice. Recent research has utilized deep neural networks for video analysis [50]. Integrating video EEG and AI into neonatal care, especially in intensive care unit settings, can detect subtle neurological events in newborns and predict cerebral dysfunction from video data alone, enhancing diagnosis and treatment [51,52,53]. Selected models for seizure prediction are shown in Table S4.

### 3.4. Epileptic Syndrome Classification

Unlike an epileptic seizure, an epilepsy syndrome is characterized by a collection of specific epilepsy-related features that typically occur together [54]. There are more than 20 known childhood epilepsy syndromes, and accurately classifying them provides vital guidance for diagnosis and treatment. Precise identification of an epileptic syndrome can provide useful information for treatment and prognosis [55]. However, a specific epilepsy syndrome could only be defined at initial diagnosis in around one-third of the children [56]. Research on AI aiding epilepsy syndrome classification is much less developed than seizure prediction and epilepsy diagnosis. Current research is typically based on interictal EEG as an analytical and learning tool for the clinical auxiliary diagnosis of epilepsy syndromes. Cao et al. [7] built a novel two-stream 3D attention module-based deep network (TSA3-D) using interictal EEG features for detecting seven childhood epilepsy syndromes, achieving an overall accuracy of 99.52%. Cui found that EEG mean, skewness, and Q_0.95_ exhibit distinctive distributions among seven typical childhood epilepsy syndromes, and a ResNet50 transfer learning model enhanced with feature selection accurately classifies these syndromes [57]. Cui et al. also presented a novel feature fusion model based on the deep transfer learning and the time–frequency representation of the scalp EEG is developed for the classifying for benign childhood epilepsy with centro-temporal spikes (BECT) and the infantile spasms with an average of 92.35% accuracy [58]. Machine learning with multiscale recurrence quantitative analysis curves from EEG can distinguish absence epilepsy from healthy control (accuracy 1.0) and autism (accuracy 0.75) [59]. Recent studies highlight incremental progress in leveraging AI to analyze non-classical EEG signatures. Myers et al. [60] developed EpiScalp, a tool that identifies epilepsy in visually normal EEGs using network fragility and source-sink connectivity metrics, bypassing reliance on overt epileptiform discharges. This approach demonstrates the potential of AI to uncover latent abnormalities in cases where traditional visual analysis fails. Similarly, Porat Rein et al. [61] pioneered ontology-driven subtyping ofBECTs, employing data mining to stratify atypical variants (e.g., Landau–Kleffner syndrome) based on EEG localization and spike morphology. Their work underscores the utility of computational frameworks in refining diagnostic granularity for poorly defined subtypes. However, lack of large-scale validation and non-motor seizures reduce its sensitivity.

AI-assisted epilepsy classification remains in its early stages, relying on limited single-hospital datasets. Expanding to larger, more diverse datasets and conducting large-scale validation are essential. Additionally, integrating clinical symptoms and seizure videos, aligned with epilepsy-specific characteristics, is vital for enhancing diagnostic accuracy beyond single-method approaches [62,63,64].

### 3.5. Epilepsy Surgery Planning

Surgery is the most cost-effective option for patients with drug-resistant focal epilepsy when a clearly identified epileptogenic zone can be effectively resected or disconnected [65]. Evaluating the Seizure Onset Zone (SOZ) is crucial for the success of epilepsy surgery. However, the current determination of the SOZ heavily relies on clinicians’ experience and their subjective interpretation of EEG, clinical symptoms, and imaging, resulting in surgical misdiagnosis rates of around 20–30% [9,66]. To enhance accuracy, computer-assisted methods are being increasingly utilized. Artificial intelligence is extensively used in EEG for epilepsy surgery, aiding in localizing seizure foci with increased safety margin, surgical efficiency, and accuracy [67]. When non-invasive preoperative evaluations including vEEG are insufficient to identify the SOZ, invasive EEG recordings using subdural or deep electrodes become necessary. Both techniques are symptom-based, aiming to accurately map the brain networks involved during seizures by considering their clinical, electrical, and anatomical relationships [68]. During preoperative evaluation, the detection of high-frequency oscillations (HFOs) (80–500 Hz) in EEG reflecting synchronized neuronal activity, have emerged as superior biomarkers for localizing SOZthan traditional epileptic discharge, considered epileptogenic biomarkers [69,70,71]. Machine learning techniques have been predominantly employed to detect SOZ in iEEG or Stereoelectroencephalography (SEEG, a type of iEEG) likely due to the scarcity of available data [72,73,74,75,76,77]. Functional connectivity analysis from scalp EEG increasing surgical candidacy even in the absence of interictal epileptiform discharges [78]. Encouragingly, some of them have been deployed in clinical settings and changed the previous resection protocols [79]. Accurate SOZ localization using CII model achieves 82.18% accuracy and strong generalizability in drug-resistant epilepsy surgery. However, consistency with clinical SOZ decreases in higher Engel classes [80]. Similarly, the Epidemic Spreading Seizure and Epilepsy Surgery framework model exhibited a comparable trend, suggesting increased seizure network complexity and complicated etiology such as multiple seizure onset zones or discordant information from different modalities [81]. Uneven distribution of the majority (non-Epileptogenic Zone (EZ)) and minorityEZ classes and large-amount, high-dimensionality characteristics of SEEG data can also strongly limit and challenge the classification performances of machine learning [82,83]. Recently, deep machine learning methods have made significant breakthroughs in processing images, speech, video, and audio, offering superior performance over traditional techniques, and overcoming current limitations through multimodal integration. Studies utilizing deep learning methods to detect epileptiform activity on scalp EEG is growing [84,85]. Utilizing higher-order statistics (HOS) and a bi-LSTM model, Sharma achieved 99.76% accuracy in classifying intracranial EEG signals from the large Bern Barcelona database, demonstrating significant clinical potential for improving epilepsy diagnosis and surgical planning [86]. Combining IEEG or SEEG with multimodal planning systems and robotic devices enables epilepsy surgery teams to enhance safety margins, surgical efficiency, and accuracy [87]. IEEG-recon was created to automate electrode reconstruction on MRI with high accuracy and speed. Validated across multiple epilepsy centers, it integrates seamlessly into clinical workflows, enhancing epilepsy surgery planning and facilitating global clinical application [88]. IEEG is limited to specific brain regions. By simultaneously combining scalp EEG and iEEG measurements in a unified multimodal approach, whole-brain electrophysiological networks can be estimated, enabling the identification of SOZ and their key transmission pathways at the whole-brain source level [89]. The Multi-center Lesion Detection algorithm, a deep learning-based MRI lesion identification tool, has effectively identified focal cortical dysplasia in MRI-negative pediatric patients with refractory focal epilepsy undergoing SEEG, achieving an accuracy of 79.2% [90].

### 3.6. Prognosis and Outcome Prediction

Electroencephalography (EEG) and neuroimaging results are the important clinical variables for predicting prognosis. AI techniques have also been applied to medical decision-making and outcome prediction in epilepsy. These applications include selecting the appropriate anti-seizure medications (ASMs) in the initial treatment, predicting individual drug responses, determining whether to reduce or discontinue medication, assessing candidacy for surgical intervention, and forecasting surgical outcomes [91,92]. In most studies, EEG is employed as one of the predictive factors combined with clinical or imaging data to predict the drug treatment [93,94,95,96,97], epilepsy commodities [98], and epilepsy surgery outcomes [99]. Despite challenges in clinical practice implementation, significant efforts have been made in recent years to develop and integrate more practical artificial intelligence tools. EEG records electrical activity directly from the cortex with relatively high spatial and temporal resolution, enabling the assessment of functional connectivity within the epileptic brain. Hence, Tomlinson et al. [100] proposed that functional connectivity networks characterized by global synchrony and local signal variability to reliably distinguish between seizure-free and persistent seizure outcomes in 17 pediatric iEEG patients after surgery. Functional connectivity features derived from scalp EEG data were also found to successfully predict vagus nerve stimulation efficacy in pediatric epilepsy [101].

Focusing on spectral analysis of different frequency bands, Sonoda et al. use machine learning and intracranial EEG spectral responses during naming tasks to predict neuropsychological outcomes after epilepsy surgery. High gamma augmentations effectively forecast postoperative language declines [102]. Jaoude introduced HEAnet, a deep learning algorithm that detects hippocampal epileptiform activity in scalp EEGs, which often goes unnoticed by human experts. This method enhances the sensitivity of scalp EEGs for diagnosing temporal lobe epilepsy. Additionally, HEAnet can assess cognitive impairments associated with epilepsy and evaluate treatment responses [84]. This contributes to more personalized and effective treatment strategies. Furthermore, Zeng et al. [103] extracted temporal and spectral domain features from to propose a reliable method to determine seizure response of KCNQ2 epileptic encephalopathy with the classification accuracy of 81.25%. Early assessment of drug responsiveness enables timely intervention, improves outcomes, and reduces risks of misdiagnosis and unnecessary burdens. Computer-aided systems have also been applied successfully in predicting seizure recurrence after AED withdrawal [104]. The integration of AI models in enhancing EEG-based prognoses serves as a quintessential example of precision medicine by tailoring treatment strategies to individual patient profiles.

### 3.7. AI Aiding Closed-Loop Seizure Suppression

Closed-loop neuromodulation is increasingly gaining attention in clinical applications such as seizure control, offering advantages over traditional open-loop methods by dynamically adjusting stimulation parameters based on real-time neural feedback. Recent advances integrate intelligent control models for predetermined actuation strategies for seizure control. For instance, the Koopman–model predictive control (MPC) framework leverages a data-driven linear dynamical model derived from the Koopman operator to predict epileptic EEG dynamics and optimize stimulation inputs viaMPC [105,106]. Similarly, a Lyapunov-based intelligent controller embedded with adaptive artificial neural networks was applied to an Izhikevich neuron network modeling the amygdala [107]. The controller utilized local field potentials as feedback to modulate network-wide synchronization, effectively suppressing ictogenesis across four distinct seizure-inducing scenarios. These models highlight the potential of combining nonlinear control theory with machine learning to compensate for unmodeled disturbances and interpatient variability. While these frameworks remain in experimental stages—tested primarily in silico or in animal models—they offer promising pathways for clinical translation. Intelligent closed-loop systems represent a paradigm shift toward personalized, adaptive neurotherapies, potentially revolutionizing the management of drug-resistant epilepsy. Future directions may extend these strategies to noninvasive modalities like transcranial magnetic stimulation or vagus nerve stimulation, broadening their therapeutic scope.

## 4. Challenges of EEG

Navigating machine learning research is complex due to multiple development and validation phases. Importantly, the use of AI in medicine remains in its nascent phase, with most efforts focused on proof-of-concept studies rather than clinical deployment. Even more “established” models, such as SCORE-AI [108] and Modified VGG-C neural network model [109] to detect interictal epileptiform discharges, are designed to mimic, match, and support expert human performance rather than as standalone independent clinical tools that can replace the expert. These methodological innovations have advanced predictive modeling capabilities, but several critical constraints still warrant careful examination.

### 4.1. Dataset Bias and Representativeness

The performance of seizure detection algorithms is inherently constrained by the characteristics of EEG datasets, including sample size, seizure-type diversity, and demographic representativeness. The imbalance between limited sample sizes and high-dimensional feature spaces, substantially increasing overfitting risks and inflating performance metrics and potentially compromise the accuracy of generalization error estimation [110]. For instance, Zhu et al. [111] observed increased model efficacy on smaller, less diverse datasets, underscoring the critical role of data scale and heterogeneity in training robust AI systems. Wearable absence seizure detection models [112] and EpiScalp [60] models to diagnose epilepsy from functional seizure face clinical limitations due to narrow applicability to specific seizure subtypes and lack generalizability across broader epilepsy classifications. Widely used repositories such as CHB-MIT, which predominantly include pediatric populations, introduce age-related biases that limit their applicability to adult epilepsy cases. Current AI-EEG methodologies also exhibit limited generalizability to atypical EEG patterns. Quon et al. [113] demonstrated high efficacy in detecting intracranial IEDs with typical morphologies (F1 score: 0.95), yet sensitivity drastically declined to 66% for atypical IEDs. This bias is further evident in Lam et al. [114], where coherence-based detection of deep mesial temporal seizures—analogous to residual epileptic activity in structurally altered brains—relied on intracranial validation (foramen ovale electrodes) and exhibited frequent false alarms (0.31/day), particularly misclassifying physiological sleep–wake transitions as seizures. Such limitations highlight the challenges in distinguishing pathological from physiological dynamics in complex scenarios, a critical issue in populations with atypical EEG patterns.

### 4.2. External Validation Deficits and Real-World Generalizability: A Notable Issue Is the Variability in Gold Standards

The absence of standardized labeling protocols and evaluation frameworks critically undermines the reproducibility and cross-study comparability of epileptic EEG research. A central manifestation of this issue lies in expert annotations, which are frequently regarded as the “gold standard” despite substantial interobserver variability. In cases of disputed IEDs, Tjepkema-Cloostermans [109] documented alarmingly low inter-rater agreement (Fleiss’ κ = 0.13), underscoring the inherent subjectivity in EEG interpretation. Such discrepancies among experts, compounded by inconsistent labeling practices, may significantly alter research outcomes [115]. Numerous factors can influence the rater in identifying a waveform as epileptiform. Clinical settings present diverse conditions. For instance, not all spike waves represent epileptiform events; their interpretation must consider the patient’s age, timing, polarity, and medical history. Waveforms may be interpreted differently by the same EEG reader if examined in a single channel vs. an entire montage or in the context of a brief two-second epoch vs. a prolonged study. The veracity of training and testing dataset labels should be scrutinized. Another fundamental barrier to clinical translation stems from insufficient external validation, compounded by prevalent reliance on intra-patient validation protocols. This methodological limitation is further exacerbated by inconsistent evaluation frameworks across studies. Researchers frequently employ disparate validation approaches—from patient-specific data partitioning to cross-institutional testing—without adhering to standardized reporting criteria. Such heterogeneity artificially inflates model performance metrics when compared to clinically relevant cross-patient or multicenter validation paradigms. For instance, Shafiezadeh et al. reported that the accuracy of an XGBoost classifier for predicting epileptic seizures dropped from 80% to 50% when tested with a more rigorous validation dataset [116]. Such overfitting risks are amplified in deep learning architectures, where CNNswith excessive parameters struggle to generalize from limited EEG trials [117,118].

### 4.3. Research Gap and Ethical Barriers

Numerous challenges persist. A critical challenge in advancing automated seizure detection lies in the mismatched priorities between clinical and engineering research, particularly in data sourcing and research priorities.

Clinical studies prioritize real-world applicability, leveraging heterogeneous datasets from multicentric trials that capture diverse epilepsy subtypes, and real-world artifacts. In contrast, engineering research predominantly relies on curated, noise-free datasets (e.g., Bonn University’s single-channel recordings), optimized for algorithmic performance but lacking ecological validity. This disconnect creates a translational chasm: models trained on idealized data fail to generalize to clinical variability, while clinically validated tools may sacrifice sensitivity for practicality. This disconnect makes the eventual clinical implementation of AI-based solutions exceedingly difficult.

The harmonious integration of AI into healthcare requires addressing ethical dilemmas [119,120,121]. Designing AI capable of performing moral tasks like humans necessitates proper training and continual refinement of its cognitive mechanisms. Also importantly, ethical concerns in AI-assisted EEG diagnostics include data privacy, as improper handling of private clinical information risks breaches and leakage, and sensitive data often lack legislative protection, making informed consent essential. Additionally, algorithm transparency poses challenges, since physicians may not fully understand AI algorithms, hindering informed decision-making and respecting patient autonomy. Furthermore, accountability remains unclear, with the consequences of AI-patient interactions potentially falling solely on patients without defined legal responsibility. Before AI can be widely adopted in clinical settings, it must adhere to machine ethics, enabling it to act as an agent of human morality. Addressing these ethical issues is crucial for the responsible and effective deployment of AI in clinical EEG diagnostics, ensuring that patient rights and safety are upheld.

## 5. Future Research Directions

To advance AI-EEG applications in epilepsy, strategic priorities should guide future efforts. First, curating clinically representative datasets is essential to overcome demographic and seizure-type biases. Collaborative initiatives must aggregate multi-center EEG data spanning age groups, comorbidities, and atypical seizure patterns such as the EEG pattern after epilepsy surgery, complemented by synthetic data strategies to address rare subtypes. Second, redefining validation standards will bridge the clinical-translational gap. Rigorous external validation protocols should integrate real-world artifacts, longitudinal assessments, and cross-institutional testing, thereby hybrid human–AI annotation systems can standardize labeling and reduce expert subjectivity. Finally, current AI systems over-rely on isolated EEG analysis, which inadequately addresses persistent artifacts caused by physiological movements (e.g., chewing, blinking) and environmental noise. While Independent Component Analysis (ICA)/Blind Source Separation (BSS) methods [122,123,124,125] and video monitoring effectively identify artifacts, their clinical adoption is hindered by high computational costs (>50% longer processing times vs. raw EEG) and poor real-time performance. Lightweight architectures integrating EEG with video/clinical data could address these limitations through synchronized artifact correction and contextual interpretation. Enhancing the integration of AI with multimodal data, including video recordings, is a critical area for improvement in model performance [49]. AI-EEG systems must adhere to ethical-legal constraints through privacy-aware training frameworks to ensure secure handling of sensitive patient data.

## 6. Conclusions

AI-enhanced EEG applications have transformed epilepsy management by enabling more accurate seizure prediction, efficient detection, precise syndrome classification, and improved surgical planning. These advancements facilitate personalized treatment and better patient outcomes. Nonetheless, significant obstacles remain, including data variability, limited dataset diversity, and ethical concerns surrounding privacy and algorithm transparency. To fully harness AI’s potential, future research must prioritize the development of robust, scalable models validated across diverse populations and integrate multimodal data sources. Additionally, establishing ethical frameworks and fostering collaboration between technologists and clinicians are essential to ensure the responsible and effective implementation of AI in clinical epilepsy care.

## Supplementary Materials

The following supporting information can be downloaded at: https://www.mdpi.com/article/10.3390/jcm14124270/s1, Table S1: Summary of EEG Databases; Table S2: Performance Metrics and Formulas; Table S3: Selected EEG-Based Seizure Prediction Models; Table S4: Selected EEG-Based Seizure Detection Models.
