# Supplementary material for: Advancements and Challenges of Artificial Intelligence-Assisted Electroencephalography in Epilepsy Management
Source: J Clin Med. 2025 Jun 16;14(12):4270. doi: 10.3390/jcm14124270 (PMC12194755; doi:10.3390/jcm14124270)
Supplement: Supplementary file 1 [file jcm-14-04270-s001.zip › jcm-3524446-supplementary.pdf]

Table S1. Summary of EEG Databases

Database	Patient Demographics	Technical Specifications
CHB-MIT Scalp EEG	22 pediatric patients with drug-resistant epilepsy (5 months–22 years); ictal/interictal data	Electrodes: 23 channels (10-20 system); Sampling: 256 Hz; file duration: mostly one hour in duration
Bonn University EEG	5 subsets (A–E): healthy subjects (A, B) and epilepsy patients (C–E; interictal/ictal recordings)	Electrodes: 128-channel system (subset-dependent); Sampling: 173.61 Hz; File duration: 23.6 seconds per segment
TUH EEG Seizure Corpus (TUSZ) dataset	300 subjects	Electrodes: 32 channels system; Sampling: 256 Hz; file duration:600 seconds
SH-SDU EEG Dataset	10 epilepsy patients (long-term monitoring); expert-annotated seizure timestamps	Electrodes: 18 channels (Fp1, Fp2, F3, F4, C3, C4, P3, P4, O1, O2, F7, F8, T3, T4, T5, T6, A1, A2); Sampling: 256 Hz; 198 hours of recordings for 191 seizure events
UCI Epileptic Seizure Recognition	10 (5 healthy, 5 epilepsy patients with temporal lobe epilepsy)	10-20 system electrodes: Single-channel (subset-dependent); Sampling: 178 Hz; file duration:

		23.6 seconds
Siena Scalp EEG database	14 epilepsy patients(9 males and 4 females)	Electrodes: 10-20 system<br>Sampling: 512 Hz;

Table S2. Performance Metrics and Formulas

Metric	Formula	Significance
Accuracy	$\frac{TP + TN}{TP + TN + FP + FN}$	Overall correctness; unreliable for imbalanced data.
Precision	$\frac{TP}{TP + FP}$	Reliability of positive predictions
Recall (Sensitivity)	$\frac{TP}{TP + FN}$	Ability to capture all positive cases
Specificity	$\frac{TN}{TN + FP}$	Ability to exclude all nagative cases
F1-Score	$\frac{2 * Precision * Recall}{Precision + Recall}$	Balances precision and recall for imbalanced data.
AUC-ROC	Area under receiver operating characteristic curve curve	Overall class separation; robust to class imbalance.

AUPRC	Area under precision-recall curve	Focuses on positive class performance; critical for severe imbalance.
Cohen's Kappa	$\frac{P_0 - P_e}{1 - P_e}$	Agreement beyond chance (2 raters); adjusts for class prevalence.
Fleiss' Kappa	$\frac{\bar{P} - \bar{P}_e}{1 - \bar{P}_e}$	Multi-rater agreement consistency (3+ raters).
FPR (false positive rate)	$\frac{FP}{FP + TN}$	Proportion of negatives flagged as positive; critical for specificity.
Latency	t_{end}-t_{start}	Time efficiency for real-time applications
Time in Warning(TIW)	$\frac{T_{warning}}{T_{total}}$	Measures how early a system predicts a seizure
G-Mean	$\sqrt{Sensitivity * Specificity}$	Balanced performance of sensitivity (ability to detect seizures) and specificity (ability to reject non-seizures)

Abbrevations: TP：true positive, TN：true negative, FP：false positive；FN：false negative

Table S3. Selected EEG-Based Seizure Prediction Models

Author	Year	EEG Dataset	Model	Internal Validation	External validation	Usage	Strength	Limitations
Antoine Spahr(1)	2025	384 patients using Empatica E4 accelerometer	3D-acc amplitude time series	cross-validation set (quantile 60) Sen=98%, FAR=1/6 days	Independent test set (347 patients, 49 CSs),Sen=96%, FAR=1/8 days	Seizure prediction	Tunable sensitivity via quantile aggregation	Limited generalizability to real-world environments ; Unproven real-time reliability on heterogeneous wearables
Baolian Shan(2)	2025	CHB-MIT	MultiSincNet (DL,optimal)	Acc=89.86%, Sen=90.52%, Spe=89.15%, F1-score (binary)=89.75%, F1-score (weighted)=89.83%, AUC−=95.10%	❌	Seizure prediction	Reduced parameters (86k) enable efficient deployment.	Relies on fixed preictal intervals
Sha Lu(3)	2025	NeuroVista dataset(10	CoSP Model(DL)	Sen=79%, FPR=0.15/h, AUC=76%	❌	Seizure prediction	Adaptability to variable preictal intervals (4–	Patient-specific optimal preictal intervals required

Author	Year	EEG Dataset	Model	Internal Validation	External validation	Usage	Strength	Limitations
		patients with refractory focal epilepsy)		TIW=0.27			180 min) for personalized prediction	
Boxuan(4) Wei	2024	CHB-MIT；Siena	AFC-GCN model(CNN,DL)	CHB-MIT: AUC=0.982,Acc=0.982,Sen=0.980, FPR=0.017<br>- Siena: AUC=0.971,Acc=0.968,Sen=0.977, FPR=0.042<br>Cross-Subject Validation :<br>- CHB-MIT: AUC=0.851,Acc=0.840,Sen=0.855, FPR=0.174<br>- Siena: AUC=0.840,Acc=0.834,Sen=0.800, FPR=0.132	❌	Seizure prediction	Dynamically infers brain connectivity via data-driven model, enhancing robustness against noise	Ignores causal brain network dynamics, limiting interpretability for seizure evolution
Xiaoshuang Wang(5)	2024	Freiburg, SWEC-ETHZ	Dual CNN-	Freiburg: Sen=100% (event)/88.1%	❌	Seizure prediction	Novel channel reordering strategy	Unverified on noisy/real EEG.

Author	Year	EEG Dataset	Model	Internal Validation	External validation	Usage	Strength	Limitations
		(18 patients), AES-SPC (5 dogs, 2 patients)	LSTM (DL)	(seg), FPR=0.10/h; SWEC-ETHZ: Sen=100%/99.1%, FPR=0/h; AES-SPC: Sen=90.5%/69.2% , FPR=0.47/h. Channel subsets outperformed full-channel.			reduces computational complexity while maintaining high accuracy	
Shao C(6)	2024	CHBMIT ; Kaggle	PCT-net (CNN + Transformer+ knowledge distillation ， hybrid)	CHBMIT: Forgetting (Acc=14.7%, Sen=19.7%, Spe=9.4%); Retaining (Acc=89.8%, Sen=87.2%, Spe=92.4%), Kaggle: Forgetting (Acc=20.9%, Sen=35.3%Spe=6.6%); Remaining (Acc=84.9%,	✗	Seizure prediction	First machine unlearning framework for seizure prediction and effective privacy preservation	Performance fluctuations caused by inter-patient similarity.

Author	Year	EEG Dataset	Model	Internal Validation	External validation	Usage	Strength	Limitations
				Sen=74.8%, Spe=94.9%).				
Nan Qi(7)	2024	CHBMIT ;KEPC	Effective with limited labeled data	25 labels：Sen=87.65% (CHB-MIT), 77.24% (KEPC)	❌	Seizure prediction	Reduces labeling cost via semi-supervised learning and unlabeled data utilization;Integrates EEG data augmentation for robust feature alignment.	Potential mode collapse in EEG data augmentation network affects synthetic signal diversity, patient-specific design limits cross-patient generalization.
T. Premawathi(8)	2024	CHB-MIT；Siena	Hybrid (KNN + Transfer Learning)	Acc=99.86%、Pr=99.9%、F1=99.9%	UCI-Epileptic Seizure Dataset：Acc=0.97	Seizure prediction	Achieved near-perfect metrics and outperforming traditional ML models, Patient-specific adaptability	Small Dataset limits generalizability due to insufficient sample size and patient homogeneity，cross-patient generalization remains unverified

Author	Year	EEG Dataset	Model	Internal Validation	External validation	Usage	Strength	Limitations
Hu(9)	2023	CHB-MIT	Hybrid Transformer (DL)	Optimal: sen=91.7%,FPR=0.00/h; General: Sen=77.0%, FPR=0.00/h	✗	Seizure prediction	First hybrid Transformer for epilepsy prediction achieveing no false alarms.	Wavelet decomposition may lose rhythm boundaries
Abbasi, M.U(10)	2019	Bonn University dataset	Double-layered LSTM(DL)	Binary: Acc=99.78%, Spe=98.92%, Sen=95.70%, Multi-class: Acc=94.81%, Spe=99.43%,Sen: 92.63%	✗	Seizure prediction	Novel dual-layered LSTM improves accuracy over SVM (2% gain in binary tasks)	Lower multi-class accuracy

Abbreviation：DL:deep learning, ML:machine learning, TL: transfer learning; Convolutional Neural Networks; KNN: k-Nearest Neighbors; Sen:sensitivity; Spe:specificity;TIW: Time in Warning; Pr:precision; Rc:Recall; FAR: False Alarm Rate; FDR: false detection rate; AUC: Receiver Operating Characteristic Area Under the Curve, TIW: Time in Warning; Pr:precision;Rc:Recall; SVM: Support Vector Machine; LSTM: Long Short-Term Memory;IED: Interictal Epileptiform Discharge

Table S4. Selected EEG-Based Seizure Detection Models

Authors	Year	EEG Dataset	Model	Internal Validation	External validation	Usage	Strength	Limitations
Mansilla D(11)	2024	104 patients	Score AI	none	acc=92% vs 94%; sen=88% vs 90%; spe=94% vs 96%; κ=56 (expert-model), κ=70 (inter-expert)	Automated routine EEG interpretation	Generalizability across populations/equipment; performance equal to experts; blinded analysis	Limited to adults (>15 years); untested in ICU/neonatal settings; retrospective design
Lin N(12)	2024	484 patients	vEpiNet (DL)	AUROC: vEpiNet=0.9902 vs. nEpiNet=0.9878 Sen(90%): vEpiNet (spe=98.8%) vs. nEpiNet (Spe=98.5%) Pr: vEpiNet=99.8% vs. nEpiNet=99.5%	AUPRC: vEpiNet=0.8623 vs. nEpiNet=0.8316 Sensitivity (80%): vEpiNet (pre=76.6%) vs. nEpiNet (pre=70.0%) FPR: vEpiNet=0.045/min vs. nEpiNet=0.063/min	IED detction	First video-EEG fusion; reduced false positives; real-time processing	Limited pediatric/infant representation

Authors	Year	EEG Dataset	Model	Internal Validation	External validation	Usage	Strength	Limitations
Tala Abdallah(13)	2025	UCI & CHU Angers datasets	DEGM model (Hybrid)	Outperforms traditional clustering, deep clustering, and supervised DL methods	❌	Seizure detection	Good model performace	High computational load for real-time deployment
Ahmed Abdelhameed(14)	2021	CHB-MIT	2D-SDCAE + Bi-LSTM (DL)	Acc= 98.79%, Sen=98.72%, Spe=98.86% ; Pr=98.86%, F1=98.79%	❌	Seizure detection	Unified supervised autoencoder for joint feature learning and classification	Limited to pediatric patients; potential hardware constraints for real-time deployment.
Ahmed M. Abdelhameed(15)	2019	Bonn University; Boston Children's Hospital	1D Convolutional VAE (DL)	Bonn: 2-class (A/E): Acc=100%, Sen=100%, Spe=100%; 3-class (A/D/E): Acc=99%, Sen=99%, Spe=99.5% Boston: Ictal vs.	❌	Seizure detection	Eliminates manual feature extraction via semi-supervised variational autoencoder	Performance variability across patients

Authors	Year	EEG Dataset	Model	Internal Validation	External validation	Usage	Strength	Limitations
				Inter-ictal: Acc=99.4% (combined 4 patients); Reduced accuracy with added patients				
Abou Jaoude M(16)	2022	Data set 1: Massachusetts General Hospital (MGH, 51 TLE patients); Data set 2: MGH (24 TLE + 20 HC); Data set 3: Brigham and Women's Hospital (BWH, 22 TLE + 24 HC)	HEAnet (CNN ensemble, DL)	(Data set 1) Data set 1 (single-event detection): AUC ROC = 0.89; AUC PRC = 0.39;	Data set 2 (TLE vs HC): AUC ROC = 0.88, lateralization Acc = 100%; Data set 3 (TLE vs HC):AUC ROC = 0.95;PPV = 93.7%;Lateralization Acc = 92%	hippocampal epileptiform activity	Noninvasive detection of hippocampal epileptiform activity via scalp EEG; outperforms human experts	Optimized for sleep data (reduced sensitivity in awake states); Potential detection of extratemporal activity; Requires validation on larger datasets; No visual confirmation of detections.
Aboyeji(17)	2025	CHB-MIT	DCSENets (DL)	Hann (window length(WL) =1s):	❌	Seizure detection	Enhanced STFT spectrograms with	Trade-offs in time-frequency resolution at

Authors	Year	EEG Dataset	Model	Internal Validation	External validation	Usage	Strength	Limitations
				Acc=87.20% Gaussian (WL=1s): Acc=87.29%, Sen=85.36, Spe=89.24 No Taper: Acc=86.85%			Hann/Gaussian taper reduce edge effects.	larger windows limit clinical utility.
Ahmad(18)	2024	Bonn University dataset	AMV-DFL (DL/ML hybrid)	Time-frequency domain deep features: Acc=99.40%, Sen=99.10%, Spe=99.10,F-score=99.15%	❌	Seizure detection	Combines multi-view learning for complementary feature representation	Requires iterative hyperparameter tuning for optimal multi-view forest performance
Alturki FA(19)	2021	CHB-MIT	KNN (ML)	Feature extraction (Common Spatial Pattern(CSP)+ logarithmic band power(LBP)+KNN)	❌	Epilepsy diagnose(epilepsy vs normal)	Novel integration of CSP with LBP for enhanced feature separation.	Computational complexity of KNN for large-scale data

Authors	Year	EEG Dataset	Model	Internal Validation	External validation	Usage	Strength	Limitations
Hu X(20)	2020	CHB-MIT	Bi-LSTM (DL)	Acc=98.62% Sen=93.61%,Spe=91.85%, G-mean=92.66%	❌	Seizure detection	Effective handling of non-linear/non-stationary EEG via local mean decomposition(LMD)	Manual statistical features from LMD may restrict adaptability to diverse EEG patterns; sensitivity to noise in specific cases
Japaridze G(21)	2023	1-channel wearable EEG (102 patients, 364 absence seizures)	CNN (DL)	❌	Average sen=78.83%, FPR=0.53/h, F1=0.823, Deficiency rate: 4.67%,False alarms: 64.71% of patients had none	Absence seizure detection	First phase 3 validation of a wearable EEG for absence seizure detection	Heavy CNN inference may drain battery life or delay alarms in resource-constrained wearables; Exclusion of patients with head size mismatch or

Authors	Year	EEG Dataset	Model	Internal Validation	External validation	Usage	Strength	Limitations
								compliance issues
Chaosong Li(22)	2021	CHB-MIT, SH-SDU	ML (SVM)	CHB-MIT (Segment-based):Sen=97.34%,Spe=97.50%, (Event-based):Sen=98.47%,FDR=0.63/h,SH-SDU(Segment-based):Sen=93.67%,Spe=96.06%, (Event-based): sen=99.39%FDR=0.64/h	✗	Seizure detection	High accuracy across both databases	Performance variability in noisy EEG, Dependency on manual parameter tuning
Punnawish Thuwajit(23)	2022	CHB-MIT TUSZ BONN	EEGWaveNet(DL)	CHI-MIT Subject-Dependent: acc=99.39%,sen=68.94%,spe=99.25%, F1-score(binary)=65.54% Subject-	✗	Seizure detection	End-to-end design reduces preprocessing; Efficient TL capability	Suboptimal sensitivity on the TUSZ dataset raises risks of missed detections in high-variance data.

Authors	Year	EEG Dataset	Model	Internal Validation	External validation	Usage	Strength	Limitations
				Independent Acc=96.17%,Sen=56.83%, Spe=96.97%, F1-score(binary)=38.26%； Transfer Learning (TL,Fine-tuned (100% data)：Acc=96.74%,Sen=75.32%,Spe=95.96%， F1-score(binary)=53.87%				
Rashed-Al-Mahfuz(24)	2021	Bonn	Deep CNN model (VGG16 or ResNet50) (DL)	(Method-3 ,FT-VGG16 classifier and continuouswavelet transform (CWT)) the highest average acc=99.21%,	✗	Seizure detection	SHapley Additive exPlanation(SHAP) analysis identifies high-frequency components (30-60Hz) as critical for	Potential noise amplification in high-frequency bands in CWT

Authors	Year	EEG Dataset	Model	Internal Validation	External validation	Usage	Strength	Limitations
				Sen=99.04%,Spe=99.38%			seizure detection	
Myers(25)	2025	Multicenter (Total: 218 patients) - Epilepsy: 99 - Non-Epilepsy: 119 (primarily Functional Seizures)	EpiScalp(ML)	AUC: 94.0% Acc=90.4% Sen=83.5% Spe=96.3%	(Independent Test Set, n=20): - Accuracy: 80% (16/20)	Epilepsy diagnosis from normal EEGs	Clean Study Labels, Detects epilepsy in normal EEGs without interictal epileptiform discharges. Interpretable model framework. Robust to demographic/clinical variations	Excluded syncope, focused on Functional Seizures.
Newton(26)	2025	Proprietary dataset from REMI sensors (4-channel wearable EEG , 50 participants)	XGBoost classifier(ML)	Event-level Sen=86.2% FDR=0.162/h	✗	Wearable seizure detection	Robust performance across age groups and generalized seizures	ambulatory noise, incomplete seizure coverage

Authors	Year	EEG Dataset	Model	Internal Validation	External validation	Usage	Strength	Limitations
Andreea M Pavel(27)	2020	Multicenter neonatal EEG data	ANSeR (ML)	Sen: 66.0% (algorithm) vs 45.3% (non-algorithm) Spe: 84.4% (algorithm) vs 89.1% (non-algorithm) FDR: 36.6% (algorithm) vs 22.7% (non-algorithm)	❌	Real-time neonatal seizure detection	Real-time detection and Enhanced Seizure Hour Detection	validation restricted to experienced centers
Peilin Zhu(28)	2024	CHB-MIT, SH-SDU	SE-TCN-BiGRU (DL)	CHB-MIT: - Patient-specific: Acc=98.77%,Sen=95.88%, Spe=99.44%, F1=96.77%, MCC=96.08% - Cross-patient: Acc=93.78%,Sen=93.31%,Spe=92.65%,F1=85.5	❌	Seizure detection	Reduced computational complexity and Efficient Real-Time Processing	reduced robustness in cross-patient scenarios

Authors	Year	EEG Dataset	Model	Internal Validation	External validation	Usage	Strength	Limitations
				5%,MCC=81.76 %				
Zhou(29)	2024	UCI, Bonn, CHB-MIT, TUSZ	RIHANet (DL, hybrid)	UCI (binary): Acc=99.76%, UCI (5-class): Acc=97.33%;Bonn(binary, (A_E-ABC_E)):Acc=100%; Bonn (5-class): Acc=96.82% CHB-MIT: Acc=99.97% (subject-dependent), 99.57% (cross-subject) TUSZ: Acc=98.41%	❌	Seizure detection	Enables multiscale feature extraction and feature sharing across layers, enhances time-frequency feature representation.	Risk of losing critical EEG features during decomposition due to EMD's non-adaptive nature
Khan(30)	2024	CHB-MIT	FDL (Fuzzy Logic,DL)	pr=0.96(normal)/0.89(seizure), sen=0.91/0.94, F1=0.93/0.91, Acc=92.57%	❌	Seizure detection	improve Black-Box DL problem	no external validation

Authors	Year	EEG Dataset	Model	Internal Validation	External validation	Usage	Strength	Limitations
Tjepkema-Cloostermans(31)	2025	MST(50 patients with focal epilepsy, 49 patients with generalized epilepsy, and 67 controls)	Modified VGG-C neural network(DL)	Sen=82.5%, Spe=99%, FDR<0.2/min, Fleiss' κ=0.80	Hybrid κ=0.62, Disputed κ=0.13, FDR= 0.10 /min,	Seizure detection	High expert consensus	Interobserver variability in external validation

Abbreviation: DL:deep learning, ML:machine learning, TL: transfer Learning; KNN: k-Nearest Neighbors; FAR: false alarm rate, FDR: false detection rate; AUROC:areas under the receiver operating characteristic curve; AUPRC: precision–recall curves; Acc : accuracy; Sen:sensitivity; Spe:specificity;TIW: time in warning; Pr:precision;Rc:recall; WL:window length;SVM: support vector machine;Bi-LSTM: bidirectional long short-term memory; STFT:short-time fourier transfor; IED: interictal epileptiform discharges; MCC:matthews correlation coefficient; LMD: local mean decomposition;CSP: common spatial pattern; LBP: local binary pattern; IMF: intrinsic mode function;CWT: continuous wavelet transform

1. Spahr A, Bernini A, Ducouret P, Baumgartner C, Koren JP, Imbach L, et al. Deep learning-based detection of generalized convulsive seizures using a wrist-worn accelerometer. Epilepsia. 2025.
2. Shan B, Yu H, Huang Y, Xu M, Ming D. Interpretable Multi-Branch Architecture for Spatiotemporal Neural Networks and Its Application in Seizure Prediction. IEEE journal of biomedical and health informatics. 2025;29(1):235-47.
3. Lu S, Liu L, Li J, Chambers J, Cook MJ, Grayden DB. Leveraging Channel Coherence in Long-Term iEEG Data for Seizure Prediction. IEEE journal of biomedical and health informatics. 2025;Pp.
4. Wei B, Xu L, Zhang J. A Compact Graph Convolutional Network With Adaptive Functional Connectivity for Seizure Prediction. IEEE transactions on neural systems and rehabilitation engineering : a publication of the IEEE Engineering in Medicine and Biology Society. 2024;32:3531-42.
5. Wang X, Gao Z, Zhang M, Wang Y, Yang L, Lin J, et al. Combination of Channel Reordering Strategy and Dual CNN-LSTM for Epileptic Seizure Prediction Using Three iEEG Datasets. IEEE journal of biomedical and health informatics. 2024;28(11):6557-67.
6. Shao C, Li C, Song R, Liu X, Qian R, Chen X. Machine Unlearning for Seizure Prediction. IEEE Transactions on Cognitive and Developmental Systems. 2024;16(6):1969-81.
7. Qi N, Piao Y, Wang Q, Li X, Wang Y. Semi-Supervised Seizure Prediction Based on Deep Pairwise Representation Alignment of Epileptic EEG Signals. IEEE Access. 2024;12:119056-71.
8. Premavathi T, Shukla M, Jadeja R. Predicting Patient-Specific Epileptic Seizures From Scalp EEG Signals Using KNN Model With Transfer Learning. IEEE Access. 2024;12:196728-39.
9. Hu S, Liu J, Yang R, Wang Y, Wang A, Li K, et al. Exploring the Applicability of Transfer Learning and Feature Engineering in Epilepsy Prediction Using Hybrid Transformer Model. IEEE transactions on neural systems and rehabilitation engineering : a publication of the IEEE Engineering in Medicine and Biology Society. 2023;31:1321-32.

10. Abbasi MU, Rashad A, Basalamah A, Tariq M. Detection of Epilepsy Seizures in Neo-Natal EEG Using LSTM Architecture. IEEE Access. 2019;7:179074-85.
11. Mansilla D, Tveit J, Aurlien H, Avigdor T, Ros-Castello V, Ho A, et al. Generalizability of electroencephalographic interpretation using artificial intelligence: An external validation study. Epilepsia. 2024;65(10):3028-37.
12. Lin N, Gao W, Li L, Chen J, Liang Z, Yuan G, et al. vEpiNet: A multimodal interictal epileptiform discharge detection method based on video and electroencephalogram data. Neural networks : the official journal of the International Neural Network Society. 2024;175:106319.
13. Abdallah T, Jrad N, El Hajjar S, Abdallah F, Humeau-Heurtier A, El Howayek E, et al. Deep Clustering for Epileptic Seizure Detection. Ieee Transactions on Biomedical Engineering. 2025;72(2):480-92.
14. Abdelhameed A, Bayoumi M. A Deep Learning Approach for Automatic Seizure Detection in Children With Epilepsy. Frontiers in computational neuroscience. 2021;15:650050.
15. Abdelhameed AM, Bayoumi M. Semi-Supervised EEG Signals Classification System for Epileptic Seizure Detection. IEEE Signal Processing Letters. 2019;26(12):1922-6.
16. Abou Jaoude M, Jacobs CS, Sarkis RA, Jing J, Pellerin KR, Cole AJ, et al. Noninvasive Detection of Hippocampal Epileptiform Activity on Scalp Electroencephalogram. JAMA neurology. 2022;79(6):614-22.
17. Aboyeji ST, Ahmad I, Wang X, Chen Y, Yao C, Li G, et al. DCSENets: Interpretable deep learning for patient-independent seizure classification using enhanced EEG-based spectrogram visualization. Computers in biology and medicine. 2025;185:109558.
18. Ahmad I, Liu Z, Li L, Ullah I, Aboyeji ST, Wang X, et al. Robust Epileptic Seizure Detection Based on Biomedical Signals Using an Advanced Multi-View Deep Feature Learning Approach. IEEE journal of biomedical and health informatics. 2024;28(10):5742-54.
19. Alturki FA, Aljalal M, Abdurraqeeb AM, Alsharabi K, Al-Shamma'a AA. Common Spatial Pattern Technique With EEG Signals for Diagnosis of Autism and Epilepsy Disorders. IEEE Access. 2021;9:24334-49.
20. Hu X, Yuan S, Xu F, Leng Y, Yuan K, Yuan Q. Scalp EEG classification using deep Bi-LSTM network for seizure detection. Computers in biology and medicine. 2020;124:103919.
21. Japaridze G, Loeckx D, Buckinx T, Armand Larsen S, Proost R, Jansen K, et al. Automated detection of absence seizures using a wearable electroencephalographic device: a phase 3 validation study and feasibility of automated behavioral testing. Epilepsia. 2023;64 Suppl 4:S40-s6.

22. Li C, Zhou W, Liu G, Zhang Y, Geng M, Liu Z, et al. Seizure Onset Detection Using Empirical Mode Decomposition and Common Spatial Pattern. IEEE transactions on neural systems and rehabilitation engineering : a publication of the IEEE Engineering in Medicine and Biology Society. 2021;29:458-67.
23. Thuwajit P, Rangpong P, Sawangjai P, Autthasan P, Chaisaen R, Banluesombatkul N, et al. EEGWaveNet: Multiscale CNN-Based Spatiotemporal Feature Extraction for EEG Seizure Detection. IEEE Transactions on Industrial Informatics. 2022;18(8):5547-57.
24. Rashed-Al-Mahfuz M, Moni MA, Uddin S, Alyami SA, Summers MA, Eapen V. A Deep Convolutional Neural Network Method to Detect Seizures and Characteristic Frequencies Using Epileptic Electroencephalogram (EEG) Data. IEEE journal of translational engineering in health and medicine. 2021;9:2000112.
25. Myers P, Gunnarsdottir KM, Li A, Razskazovskiy V, Craley J, Chandler A, et al. Diagnosing Epilepsy with Normal Interictal EEG Using Dynamic Network Models. Annals of neurology. 2025;97(5):907-18.
26. Newton TJ, Frankel MA, Tosi Z, Kazen AB, Muvvala VK, Loddenkemper T, et al. Validation of a discrete electrographic seizure detection algorithm for extended-duration, reduced-channel wearable EEG. Epilepsia. 2025.
27. Pavel AM, O'Toole JM, Proietti J, Livingstone V, Mitra S, Marnane WP, et al. Machine learning for the early prediction of infants with electrographic seizures in neonatal hypoxic-ischemic encephalopathy. Epilepsia. 2023;64(2):456-68.
28. Zhu P, Zhou W, Cao C, Liu G, Liu Z, Shang W. A Novel SE-TCN-BiGRU Hybrid Network for Automatic Seizure Detection. IEEE Access. 2024;12:127328-40.
29. Zhou Q, Zhang S, Du Q, Ke L. RIHANet: A Residual-based Inception with Hybrid-Attention Network for Seizure Detection using EEG signals. Computers in biology and medicine. 2024;171:108086.
30. Khan FA, Umar Z, Jolfaei A, Tariq M. Explainable Fuzzy Deep Learning for Prediction of Epileptic Seizures Using EEG. IEEE Transactions on Fuzzy Systems. 2024;32(10):5428-37.
31. Tjepkema-Cloostermans MC, Tannemaat MR, Wieske L, van Rootselaar AF, Stunnenberg BC, Keijzer HM, et al. Expert level of detection of interictal discharges with a deep neural network. Epilepsia. 2025;66(1):184-94.
